# Acquisition and dissemination of cephalosporin-resistant *E*. *coli* in migratory birds sampled at an Alaska landfill as inferred through genomic analysis

**DOI:** 10.1038/s41598-018-25474-w

**Published:** 2018-05-09

**Authors:** Christina A. Ahlstrom, Jonas Bonnedahl, Hanna Woksepp, Jorge Hernandez, Björn Olsen, Andrew M. Ramey

**Affiliations:** 1 U.S. Geological Survey, Alaska Science Center, Anchorage, Alaska 99508 USA; 2 Department of Clinical and Experimental Medicine, Linköping University, Linköping, SE-58183 Sweden; 3 Department of Infectious Diseases, Kalmar County Hospital, Kalmar, SE-39185 Sweden; 4 Department of Clinical Microbiology, Kalmar County Hospital, Kalmar, SE-39185 Sweden; 5 Zoonosis Science Center, Department of Medical Sciences, Uppsala University, Uppsala, SE-75185 Sweden

## Abstract

Antimicrobial resistance (AMR) in bacterial pathogens threatens global health, though the spread of AMR bacteria and AMR genes between humans, animals, and the environment is still largely unknown. Here, we investigated the role of wild birds in the epidemiology of AMR *Escherichia coli*. Using next-generation sequencing, we characterized cephalosporin-resistant *E*. *coli* cultured from sympatric gulls and bald eagles inhabiting a landfill habitat in Alaska to identify genetic determinants conferring AMR, explore potential transmission pathways of AMR bacteria and genes at this site, and investigate how their genetic diversity compares to isolates reported in other taxa. We found genetically diverse *E*. *coli* isolates with sequence types previously associated with human infections and resistance genes of clinical importance, including *bla*_CTX-M_ and *bla*_CMY_. Identical resistance profiles were observed in genetically unrelated *E*. *coli* isolates from both gulls and bald eagles. Conversely, isolates with indistinguishable core-genomes were found to have different resistance profiles. Our findings support complex epidemiological interactions including bacterial strain sharing between gulls and bald eagles and horizontal gene transfer among *E*. *coli* harboured by birds. Results suggest that landfills may serve as a source for AMR acquisition and/or maintenance, including bacterial sequence types and AMR genes relevant to human health.

## Introduction

Antimicrobial resistance (AMR) in bacterial pathogens is a growing threat to human and animal health, with an increasing number of infections no longer responding to once-standard treatments^1,2^. Cephalosporin-resistant bacteria, including those producing extended spectrum β-lactamases (ESBLs), are particularly concerning to public health, due to their resistance to commonly prescribed beta-lactam antibiotics, their common co-resistance to other antimicrobial agents, and their increasing global prevalence^3,4^. While exposure to AMR pathogens presents obvious human and animal health risks due to the potential of contracting difficult-to-treat bacterial afflictions, non-pathogenic bacteria harbouring resistance determinants may also represent a more cryptic threat to public health given that genes conferring AMR can be transferred to bacterial pathogens via horizontal gene transfer^5^. Furthermore, bacteria and genes conferring AMR have the potential to spread and proliferate through humans, animals, and the environment^6,7^, prompting the need for a coordinated One Health approach to understand direct and indirect pathways of dissemination and to inform risk management^8,9^.

Most antibiotic compounds are naturally occurring in the environment^10^ and AMR in soil-dwelling bacteria predates antibiotic use by humans^11^. However, there is a large body of evidence that widespread use of antibiotics by humans and their application in livestock production has increased the prevalence of AMR in bacterial communities of humans, animals, and the environment^12–17^. Significant data gaps regarding the acquisition, distribution, and proliferation of AMR determinants have hampered efforts at quantifying the human health risk posed by AMR bacterial pathogens and AMR determinants from environmental sources^5^. Thus, understanding risk to human health requires active surveillance for AMR bacteria in diverse environments to elucidate transmission pathways and to provide information on the extent of horizontal gene transfer among environmental sources and human microbial communities.

Free-ranging wildlife represents one environmental source by which AMR bacteria may emerge and/or be maintained. While there is substantial support for the premise that anthropogenic inputs into the local environment^18–21^, or a relative lack thereof^16,22,23^, influence the prevalence of AMR bacteria among wildlife inhabiting an area, the role of free-ranging animals in maintaining and dispersing such bacteria is less clear^24^. Wild birds have been a common focus for investigations to understand the occurrence of AMR bacteria in the environment because many species are relatively abundant, use anthropogenically influenced habitats, and disperse over relatively long distances^25^. Thus, birds may be informative taxa for understanding the ecology of AMR bacteria and for gaining insight into proliferation and dispersal. Gulls (family *Laridae*) in particular appear to represent useful model species for such research because of their tendency to forage in response to human activities^26^, their apparent propensity to be colonized with AMR *Escherichia coli* at relatively high rates^27–30^, and circumstantial evidence indicating that some species may transport AMR bacteria through migratory movements^23,28,31^. Additionally, wild raptors may provide an alternative indicator of AMR in the environment based on their predatory nature, and thus their potential to acquire AMR bacteria harboured by diverse prey^32–34^.

The use of high resolution molecular approaches, such as next-generation whole genome sequencing (WGS), can help resolve pathways by which AMR bacteria are maintained and dispersed in different environments. Evaluation of the significance of genetically similar AMR genes between hosts and locations^35,36^ can inform risk assessments regarding AMR threats to human and animal population health^5,37^. However, attributing the source and transmission routes of AMR bacteria and AMR determinants that are clinically relevant to human and animal health is currently confounded by the paucity of molecular epidemiological data on AMR determinants outside of the clinic. Therefore, in this study, we genomically characterized cephalosporin-resistant *E*. *coli* in sympatric gulls and bald eagles inhabiting a landfill habitat to gain insight into the genetic determinants conferring AMR in these environmental sources, to explore potential transmission pathways and evolutionary mechanisms contributing to AMR at this site, and to investigate how bacterial diversity compares to isolates previously reported in other taxa and clinical settings. Results provide insight into how AMR *E*. *coli* and AMR determinants are maintained and shared among sympatric birds inhabiting an anthropogenically influenced habitat and suggest plausible sources of exposure.

## Results

### Phenotypic characterization of cephalosporin-resistant *E*. *coli* isolates

A total of 27 cephalosporin-resistant *E*. *coli* isolates were recovered from CHROMagar C3G^R^ plates, 13 and 14 of which originated from faecal samples collected from bald eagles and gulls, respectively. Phenotypic resistance was tested against 18 antibiotics, with all 27 isolates resistant to Ampicillin and Cefadroxil. Most isolates were resistant to between four and seven antibiotics (range = 4–13; median = 6), but one isolate (A1_007_Gull) was resistant to 13 antibiotics (Supplementary Table S1). According to the definition of multidrug resistance proposed by Schwarz *et al*.^38^ (i.e. bacteria exhibiting resistance to at least one agent in at least three antimicrobial classes), 11 isolates displayed phenotypic multidrug resistance.

### Whole genome sequencing and *de novo* assembly

The conservatively estimated average depth of coverage for 27 cephalosporin-resistant *E*. *coli* isolates originating from gull and bald eagle faecal samples on which WGS was performed was 29× and there was an average of 149 contigs >500-base pairs after genome assembly using SPAdes (detailed assembly metrics are provided in Supplementary Table S2). Sequencing coverage was low (an average of 7.5-fold coverage) for one bald eagle faecal isolate (A1_020_BaldEagle), which was therefore excluded from further analyses. Thus, genomic sequencing data from a total of 26 *E*. *coli* isolates originating from 14 gull and 12 bald eagle faecal samples was further analysed.

### *E*. *coli* sequence types and core genome phylogeny

**Figure 1:**
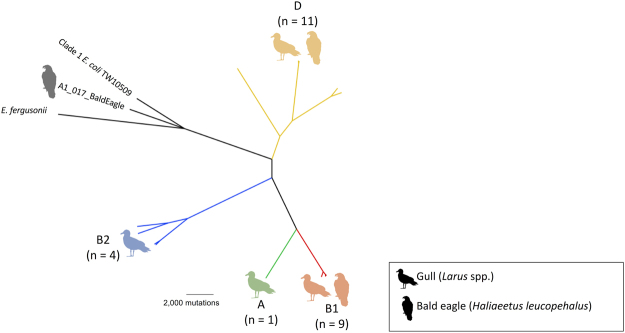
Unrooted clonal core genome phylogeny of 26 cephalosporin-resistant *E*. *coli* isolates originating from gulls and bald eagles in Alaska, *E*. *fergusonii* (NC_011740.1) and a Clade 1 *E*. *coli* sequence (TW10509; NZ_GL872204.1). Phylotypes A (green), B1 (red), B2 (blue) and D (yellow) are indicated at the tips with an image of a gull, bald eagle, or both, according to the species from which the isolates were obtained. The number of cephalosporin-resistant *E*. *coli* isolates belonging to each phylotype is indicated in parentheses. Silhouette images of gulls (credit: Rebecca Groom, https://creativecommons.org/licenses/by/3.0/legalcode) and bald eagles were sourced from PhyloPic (www.phylopic.org).

The maximum likelihood phylogeny based on the core genome (i.e. orthologous regions present in all genomes), and estimated using ClonalFrameML to account for mutation and recombination events, revealed a diverse population of *E*. *coli* isolates (Fig. 1). All four major *E*. *coli* phylotypes were identified through *in silico* phylotyping. Most isolates belonged to phylotype D, which accounted for 42% (n = 11) of isolates, including *E*. *coli* recovered from samples collected from both bald eagles (n = 4) and gulls (n = 7). Phylotypes B1, B2, and A accounted for 35% (n = 9), 15% (n = 4), and 4% (n = 1) of isolates, respectively. Phylotypes A and B2 were represented only by isolates from gulls, whereas those assigned to phylotype B1 included both isolates from bald eagles and gulls. One additional isolate, A1_017_BaldEagle, was most closely related to the reference strain *E*. *coli* TW10509, which represents a divergent, “cryptic” lineage termed Clade 1^39^.

**Figure 2:**
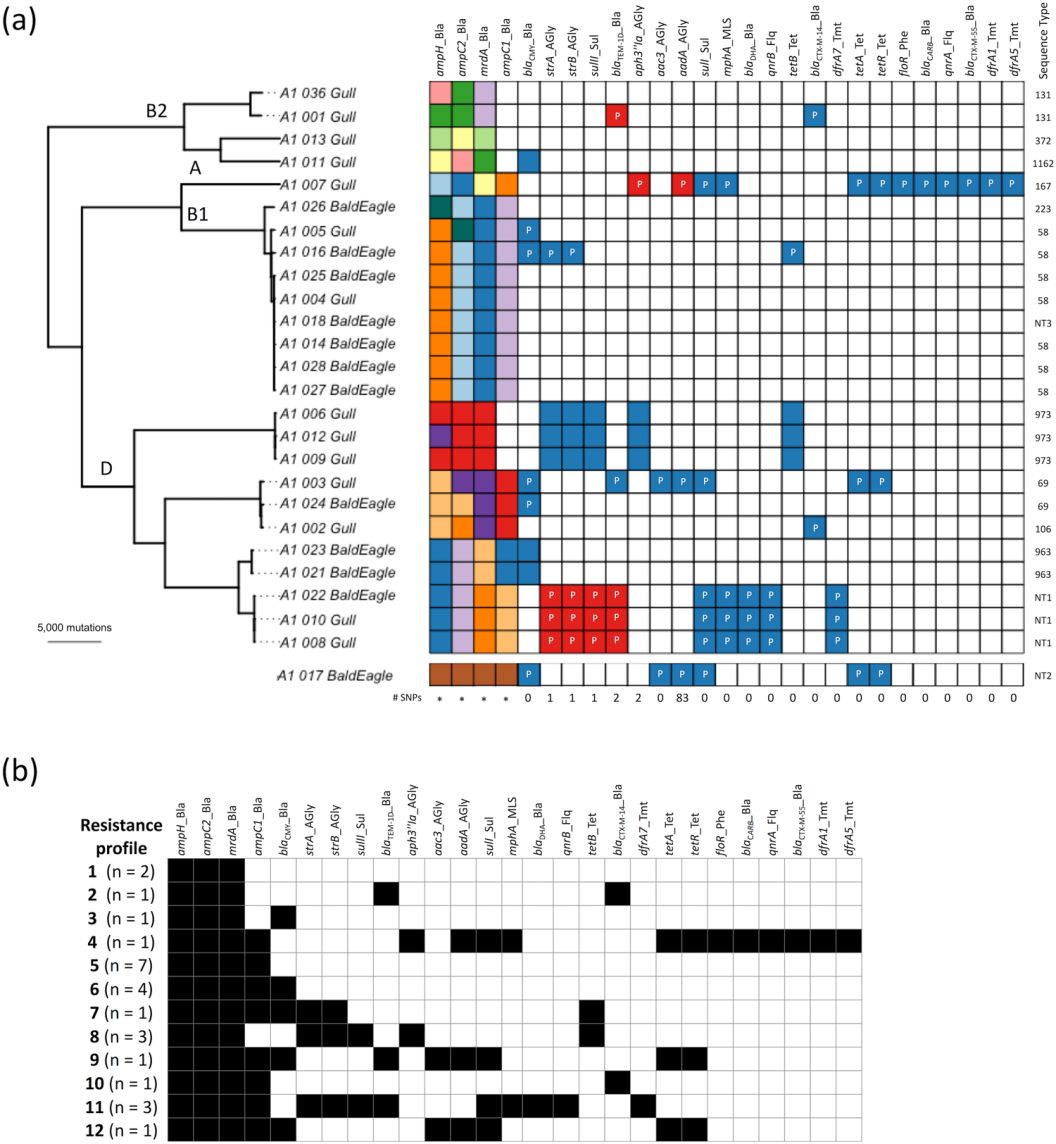
(**a**) Midpoint rooted clonal core genome phylogeny of 25 cephalosporin-resistant *E*. *coli* isolates originating from gulls and bald eagles in Alaska. Phylotypes (A, B1, B2, D) are indicated on branches. Presence of each of 27 identified AMR genes is shown as a matrix, with gene names indicated at the top of the matrix underscored by the antibiotics class (AGly: aminoglycosides, Bla: beta-lactamases, Flq: fluoroquinolones, MLS: macrolide-lincosamide-streptogramin, Phe: phenicols, Sul: sulfonamides, Tet: tetracyclines and Tmt: trimethoprim). Coloured squares indicate presence and white indicates that the gene was not found. Each colour indicates a different allele for each gene. The number of SNPs differentiating AMR genes with two alleles is shown below the matrix, while the phylogenetic diversity of genes with more than two alleles (indicated by *) can be seen in Fig. 3. AMR genes found on plasmids identified by PlasmidSPAdes are indicated with the letter “P”. The final column of the matrix indicates *in silico* identified *E*. *coli* sequence types. The divergent A1_017_BaldEagle isolate is included at the bottom of the matrix, as it was omitted from the midpoint rooted clonal core genome phylogeny. **(b)** Unique resistance profiles identified through *in silico* AMR gene detection in cephalosporin-resistant *E*. *coli* isolates. A presence/absence matrix of 27 identified AMR genes is shown, with black shading indicating presence, and no shading indicating that the gene was not found. The number of isolates found with each resistance profile is indicated in parentheses.

When reference strains and the divergent A1_017_BaldEagle isolate were excluded, a total of 176,043 SNPs were detected in the 4,734,259-base pair core genome alignment of 25 cephalosporin-resistant *E*. *coli* recovered from gulls and bald eagles. The ClonalFrameML log-likelihood ratio indicated there was evidence of recombination, with an estimated mean rate of recombination to mutation (R/θ) of 0.66. Accounting for recombination, an estimated clonal phylogeny of the 25 core genomes revealed extensive genetic diversity between small clusters of genetically closely related isolates (i.e. >99.95% nucleotide identity; Fig. 2a).

*In silico* MLST analysis identified 13 sequence types (Table 1). Sequence type 58 was the most common type, representing 27% (n = 7) of all isolates, followed by ST973 (n = 3), NT1 (n = 3), ST69 (n = 2), ST131 (n = 2), and ST963 (n = 2). The remaining sequence types, ST106, ST167, ST223, ST372, ST1162, NT2, and NT3, were represented by a single isolate each. Three new sequence types (NT) were identified, one of which was represented by three isolates. Based on the core genome, the NT3 isolate was closely related to ST58 isolates and differed from the ST58 MLST profile by a single SNP in *purA*. We compared the two ST131 isolates from gulls in our study to previously sequenced ST131 isolates that represented the three reported ST131 clades^35^. A1_036_Gull was identified as clade B and A1_001_Gull was identified as clade A, based on a separate core genome analysis using Parsnp (Supplementary Fig. S1).

**Table 1:** Genomic characteristics of 26 cephalosporin-resistant *E. coli* isolates originating from gull and bald eagle faecal samples in Alaska.

Isolate ID	Phylotype	Sequence Type	K-Pax2 cluster	# unique CDS
A1_001_Gull	B2	131	8	57
A1_002_Gull	D	106	2	34
A1_003_Gull	D	69	2	143
A1_004_Gull	B1	58	1	0
A1_005_Gull	B1	58	5	68
A1_006_Gull	D	973	3	2
A1_007_Gull	A	167	9	102
A1_008_Gull	D	NT1^†^	4	1
A1_009_Gull	D	973	3	60
A1_010_Gull	D	NT1^†^	4	2
A1_011_Gull	B2	1162	6	170
A1_012_Gull	D	973	3	103
A1_013_Gull	B2	372	6	158
A1_014_BaldEagle	B1	58	1	1
A1_016_BaldEagle	B1	58	5	36
A1_017_BaldEagle	A	NT2^†^	10	179
A1_018_BaldEagle	B1	NT3^†^	1	11
A1_021_BaldEagle	D	963	7	100
A1_022_BaldEagle	D	NT1^†^	4	65
A1_023_BaldEagle	D	963	7	99
A1_024_BaldEagle	D	69	2	9
A1_025_BaldEagle	B1	58	1	0
A1_026_BaldEagle	B1	223	5	0
A1_027_BaldEagle	B1	58	1	0
A1_028_BaldEagle	B1	58	1	0
A1_036_Gull	B2	131	8	0

^†^NT = new sequence type.

### Accessory genome characteristics

AMR gene detection using SRST2 identified a total of 27 different AMR genes, three of which (*ampH*, *ampC2*, *mrdA* [encoding Penicillin binding protein 2]) were found in all isolates. These three genes, as well as *ampC1* that was identified in 19 isolates, are chromosomal *E*. *coli* genes with point mutations associated with AMR. Genes conferring resistance to beta-lactams predominated (37% of genes), followed by those conferring resistance to aminoglycosides (19% of genes). One *bla*_CTX-M-55_ and two *bla*_CTX-M-14_ positive isolates were found and eight isolates harboured the pAmpC gene *bla*_CMY-2_. Twelve unique presence/absence resistance profiles were identified (Fig. 2b). Identical profiles were observed in up to seven isolates from both gulls and bald eagles and in highly genetically divergent isolates based on the core genome phylogeny (Fig. 2a). Conversely, some isolates (e.g. A1_003_Gull vs A1_024_BaldEagle or A1_002_Gull) with highly similar core genomes had very different resistance profiles. A total of 61 individual, non-intrinsic AMR genes were detected in putative plasmids assembled using PlasmidSPAdes, whereas 18 acquired AMR genes were not detected in plasmids using this method (Fig. 2a).

**Figure 3:**
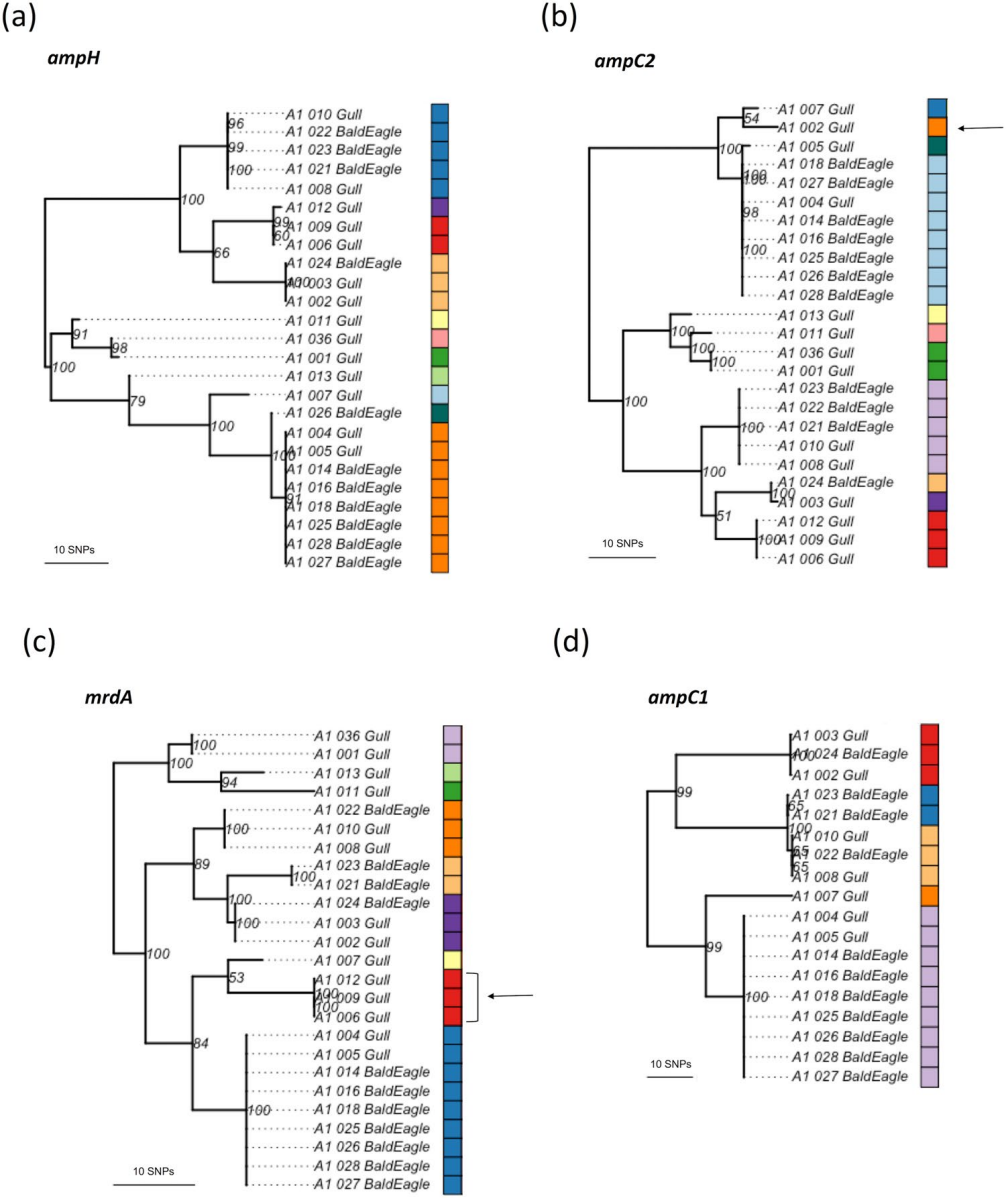
Midpoint rooted maximum likelihood phylogenies of cephalosporin-resistant *E*. *coli* isolates originating from gulls and bald eagles in Alaska based on individual chromosomally-encoded AMR genes – (**a**) *ampH*, (**b**) *ampC2*, (**c**) *mrdA*, and (**d**) *ampC1*. Each colour indicates a different allele for each gene sequence. Arrows indicate incongruence with the core genome phylogeny. Branch support values are indicated and based on 100 bootstrap pseudoreplicates.

We compared individual AMR gene sequences between isolates, identifying more than one genotype in 10 genes and more than two genotypes in four genes (Fig. 2a). Individual maximum likelihood phylogenies were estimated for the four intrinsic chromosomal genes with more than two genotypes (Fig. 3). In most cases, individual gene phylogenies clustered isolates similarly as compared to the core genome phylogeny; however, this did not occur on two occasions. The A1_002_Gull phylotype D *ampC2* nucleotide sequence was most closely related to a phylotype A isolate, differing by only seven SNPs. Additionally, three phylotype D isolates (A1_006_Gull, A1_009_Gull, and A1_012_Gull) were more closely related to the phylogroup A and phylogroup B1 isolates than to other phylogroup D isolates based on *mrdA*.

Biocide and heavy metal resistance genes were identified *in silico* in isolates collected from gulls and bald eagles by querying against a database of 704 experimentally confirmed resistance genes, resulting in the identification of a total of 96 different biocide/heavy metal resistance genes in our collection of 26 cephalosporin-resistant *E*. *coli* isolates. A range of 22–60 biocide and heavy metal resistance genes were found per isolate (median = 34) and the presence of individual genes ranged from detection in 1–26 isolates (median = 6.5) (Supplementary Fig. S2). Ten of the 96 biocide/heavy metal resistance genes detected among gull and bald eagle isolates are reported to be plasmid-borne, with the remainder being chromosomally encoded. The 96 biocide/heavy metal resistance genes detected were predicted to confer resistance to 64 different compounds, with resistance to hydrogen peroxide and zinc being the most common among our isolates. To investigate the possibility of plasmid co-localization of AMR genes and biocide/heavy metal resistance genes, the isolate with the highest number of resistance genes (A1_007_Gull) was investigated in detail by visualizing detected genes on the 81 PlasmidSPAdes-assembled contigs. All 12 mobile AMR gene sequences were located within an estimated five nodes (i.e. assembled contigs) of a biocide/heavy metal resistance gene, *qacEdelta1*, which was located on the same node as the AMR gene *SulI* (Supplementary Fig. S3). Additionally, we identified the class 1 integron-integrase gene *intI1* in three assemblies (A1_003_Gull, A1_007_Gull, and A1_017_BaldEagle) using *in silico* PCR amplification.

LS-BSR identified 3,487 core, 7,332 accessory, and 1,400 unique (i.e. present in a single genome) CDS among all 26 genomes (Table 1). Isolate A1_017_BaldEagle had the largest number of unique CDS (n = 179). K-Pax2 partitioned isolates into ten distinct clusters based on the presence/absence of 1,844 accessory CDS that were determined to be significantly discriminatory (Table 1). The number of isolates per cluster ranged from one to six and isolates were distributed among clusters similarly to the topology inferred from the core genome phylogeny.

## Discussion

We genomically characterized 26 cephalosporin resistant *E*. *coli* isolates from gulls and bald eagles sampled at a landfill in southcentral Alaska. The clonal core genome phylogeny of the major *E*. *coli* phylotypes identified in our isolates, as well as the divergent Clade 1 bald eagle isolate, was consistent with the general branching pattern of phylogenetic trees previously reconstructed for *E*. *coli* isolated from humans and animals^40,41^. However, we found a predominance of phylotype D *E*. *coli* isolates among our sample collection, followed by B1 isolates, whereas previous studies reported that B1 strains predominated in domestic and wild animals^40^, and specifically in birds^42^. This may be attributed to our culture methodology that selected for cephalosporin-resistant *E*. *coli*. Previous observations found that phylotypes A and D were more permissive to develop resistance to third-generation cephalosporins^40^, which is further supported in our study where phylotype A and D isolates generally harboured a higher number of AMR genes compared to phylotypes B1 and B2. The core genome of phylotype B1 isolates in our study showed the lowest degree of genetic diversity, as has been previously reported^42^.

We recovered closely related cephalosporin-resistant *E*. *coli* isolates from faecal samples of bald eagles and gulls inhabiting the same landfill, including two groups of nearly genetically indistinguishable *E*. *coli* isolates. The finding that five bald eagles and one gull harboured *E*. *coli* isolates with over 99.95% core genome nucleotide identity (sequence types 58 and NT3) and one bald eagle and two gulls carried similarly genetically closely related *E*. *coli* isolates (sequence type NT1) suggests acquisition via two possible scenarios: 1) common point source (via similar foraging behaviour by gulls and eagles at the landfill or pirating behaviour) or 2) through an inter-species transmission pathway (via faecal-oral route or predation of gulls by eagles). Further efforts are needed to understand the precise mechanisms by which cephalosporin-resistant *E*. *coli* may be shared among these sympatric taxa, and the potential implications this has for the spatial dissemination of AMR enteric bacteria by wild birds.

All 10 MLST sequence types found in our study that could be assigned to an existing type have previously been isolated from humans - nine of which have caused human disease (http://enterobase.warwick.ac.uk). These 10 MLST sequence types found in gulls and bald eagles included globally widespread, clinically important *E*. *coli* sequence types, including the often-multi-drug resistant and pathogenic ST131 and ST69 clones^43–45^. ESBL-producing ST131 *E*. *coli* isolates have also been isolated from several wild and domestic animal species, including gulls in Barrow (now Utqiaġvik), Alaska^30^ and Winnipeg, Canada^46^, the latter of which carried, among others, the two *bla*_CTX-M_ genotypes found in our study (*bla*_CTX-M-14_ and *bla*_CTX-M-55_). The two ST131 isolates from our study belonged to ST131 clade A and clade B, with the clade A isolate harbouring acquired ESBL genes *bla*_CTX-M-14_ and *bla*_TEM_. This supports previous findings that *bla*_CTX-M-14_ is associated with clades A and C1 and that *bla*_CTX-M_ genes are rarely found in clade B isolates^47^. CTX-M enzymes hydrolyse a wide variety of newer generation β-lactam antibiotics^48^ and have been increasing in incidence globally. CTX-M-14 and CTX-M-15 genotypes are particularly widespread, and the prevalence of CTX-M-55 is increasing in China^49^. The isolate in our study with the highest number of resistant genes, A1_007_Gull, belonged to ST167. In clinical human derived isolates, ST167 has been found to harbour resistance genes to last-line of defense antibiotics, such as carbapenems and colistin^50^. However, we did not find evidence for resistance to these two antibiotics in the ST167 gull isolate recovered in our sample collection. Additional WGS of *E*. *coli* isolates from humans and birds, combined with MLST results, would be useful for providing further inference as to the utility of commonly employed genetic approaches (e.g., MLST) for elucidating potential epidemiological connections across the human-animal interface.

The cephalosporin-resistant *E*. *coli* sequence types and AMR gene alleles found in gulls and bald eagles in our study have previously been reported to be widespread, making it difficult to identify specific sources for these isolates and associated resistance genes. However, cephalosporin-resistant *E*. *coli* isolates were previously recovered from gull faeces at the Soldotna landfill in 2014, but were not isolated from spatially-proximate samples collected at the mouth of the Kenai River or more spatially-distant samples collected from Middleton Island^21^. Thus, it is plausible that the Soldotna landfill either serves as a point source for cephalosporin-resistant *E*. *coli* or at least provides selection pressures to maintain AMR *E*. *coli* in the environment. This inference is substantiated by previous research that found increased AMR bacteria in gulls foraging in anthropogenic environments^51^.

Cephalosporin-resistant *E*. *coli* isolates genomically characterized in our study harboured genes conferring resistance to 64 different biocide and heavy metal compounds, many of which are reported to be plasmid-borne. The presence of heavy metals and biocides in contaminated environments, such as landfills^52^, can facilitate horizontal gene transfer^53,54^ and co-select for AMR genes, since genes conferring resistance to heavy metals and antibiotics are often physically linked^55,56^. We found evidence of this in the one isolate we investigated in detail, with *qacEdelta1*, a biocide/heavy metal resistance gene, co-located with *SulI*, an AMR gene, on a plasmid. The association of these two genes has been observed previously^57,58^, and although correlative and anecdotal in the current study, the finding of co-occurrence of AMR determinants and genes conferring resistance to biocides and heavy metals among cephalosporin-resistant *E*. *coli* isolated from gulls and bald eagles is consistent with the premise that the Soldotna landfill may serve as a source for acquisition and maintenance of AMR bacteria by wild birds.

Three (11.5%) isolates, two from gulls and one from a bald eagle, harboured the class 1 integron-integrase gene, *intI1*, a target that is often linked to genes conferring AMR and that has previously been used as a proxy for anthropogenic pollution^59^. Four resistance genes (three with identical nucleotide sequences), all predicted to be plasmid-borne, were found only in these three isolates, suggesting horizontal gene transfer as a likely mechanism of dissemination of *intI1* among bacteria harboured by birds. Additional research regarding the prevalence of *intI1* in bacteria harboured by wild birds in environments differently impacted by anthropogenic inputs would be helpful for interpretation of the significance of these findings.

Phylogenetic clustering of isolates in each of the chromosomal AMR gene phylogenies generally closely matched the core genome phylogeny; however, the finding that phylotype D isolates clustered with phylotype A isolates in two of the four AMR gene sequence phylogenies is indicative of homologous recombination. Our ClonalFrameML results provides additional evidence for recombination. Contrary to our findings, previous analysis of inter-phylotype recombination found phylotype D strains more likely to recombine with phylotype B2 strains, whereas phylotype A strains were more likely to recombine with types B1 and E^41^. Nevertheless, most resistance determinants found in the isolates investigated in this study were predicted to be plasmid-mediated and therefore, we infer that horizontal gene transfer was likely more influential than homologous recombination in the distribution of resistance genes among isolates in the current study.

We classified 77% of individual AMR genes detected in our study as plasmid-borne, however, this may not reflect the true number of plasmid-encoded genes due to challenges in plasmid sequence assembly^60^. Complete plasmid sequence reconstruction from short-read sequencing data is problematic, as resistance genes are often flanked by repetitive elements making them difficult to assemble^61,62^. Interestingly, no plasmid-borne AMR genes were detected in phylotype D isolates A1_006_Gull, A1_012_Gull, and A1_009_Gull, despite all three isolates having identical resistance profiles, characterized by five acquired AMR genes. It is possible that PlasmidSPAdes failed to recognize putative plasmid sequences in these isolates, and others where plasmid-encoded genes were expected (e.g. *bla*_CMY_-positive isolates), or these genes may have been integrated into the *E*. *coli* chromosome via integrons. These findings exemplify the need for improved bioinformatics tools to investigate accessory genomes of bacteria in order to understand mechanisms by which AMR determinants may be acquired and dispersed.

Several of the AMR genes identified in our study had 100% nucleotide identity to genes found in human clinical isolates, including two (*tetA* and *sulI*) of the seven AMR genes found in soil bacteria that matched human clinical isolates reported by Forsberg *et al*.^63^. While this could be interpreted as evidence of recent transmission at the soil-human-animal interface, this could also indicate that some gene alleles are particularly widespread and/or under purifying selection pressures. Such detailed molecular characterization of AMR genes can, conceptually, help resolve the epidemiology of resistance and identify the relative importance of different sources and transmission pathways. However, bacteria harbouring identical AMR genes, or even the genes themselves, may still be unrelated on an epidemiologically-relevant timescale. Thus, increased molecular surveillance of AMR genes in multiple hosts and environments to assess the relative prevalence of AMR gene alleles in different hosts/environments is critical if we are to gain rigorous inference regarding the dissemination of AMR genes and transmission pathways.

In summary, we found evidence that cephalosporin-resistant *E*. *coli* isolates were abundant among gulls and bald eagles at a landfill in southcentral Alaska. Identified sequence types of *E*. *coli* isolates included those previously associated with human infections, as well as presence of AMR determinants of clinical importance. Furthermore, genomic analyses provided evidence that gulls and bald eagles acquired bacteria via common point sources and/or through transmission among individuals, with horizontal gene transfer likely playing some role in the evolution of *E*. *coli* resistance maintained by the taxa sampled. Without environmental sampling at the landfill and other locations frequented by gulls and bald eagles, it cannot be conclusively determined whether the landfill is a source of AMR genes, whether this habitat provides selection pressure for AMR *E*. *coli* to persist, or whether the landfill is simply a foraging site for previously colonized birds. However, our results are consistent with previous studies that support the premise that anthropogenically influenced habitats play a role in the maintenance of AMR determinants in the environment. Future work to clarify point sources of AMR *E*. *coli* in wild birds and associated transmission pathways should incorporate sampling for AMR determinants in the physical environment and through space and time to better assess the transfer of AMR genes among bacteria associated with soil, water, and wildlife.

## Materials and Methods

### Sample collection and culture

Faecal material was collected from 20 bald eagles (*Haliaeetus leucopehalus*) and 56 gulls, which included glaucous-winged gulls (*Larus glaucescens*), American herring gulls (*Larus argentatus*), and hybrids, at the Soldotna landfill in southcentral Alaska (60.448°N, 151.118°W) from 7–9 June 2016. Samples were collected by inserting a sterile swab into recently deposited faecal material (all samples from bald eagles and 50 of those from gulls) or directly into the cloaca of live-captured gulls (n = 6) caught using noose carpets (authority granted under Alaska Department of Fish and Game permit #16–109, U.S. Fish and Wildlife permit #MB789758-5, U.S. Geological Survey Alaska Science Center Animal Care and Use Committee approval #2016-6). All methods were performed in accordance with the relevant guidelines and regulations. Swabs were subsequently placed into a vial with chilled Luria broth (BD, Sparks, USA), and kept cool on ice packs, for approximately 4–48 hours until frozen at −80 °C.

For *E*. *coli* culture, samples were thawed and inoculated in 2 ml brain heart infusion broth (BHI; Becton Dickinson, USA), supplemented with vancomycin (16 mg/L; ICN Biomedicals Inc., USA) to select for gram negative bacteria, using a sterile swab. Following incubation for 18–24 hours at 36 °C for enrichment, 10 μl of broth was streaked onto CHROMagar C3G^R^ plates (CHROMagar, France), a medium that supports growth of bacteria with reduced susceptibility to third generation cephalosporins. *E*. *coli* CCUG 17620 and *K*. *pneumoninae* CCUG 45421 were included as negative and positive controls, respectively. All plates were incubated in aerobic conditions for 18–24 hours at 36 °C. Putative *E*. *coli* isolates (one per plate) were analysed by matrix-assisted laser desorption ionization time of flight mass spectrometry (Bruker Corporation, Germany), as described previously^64^ and antimicrobial susceptibility testing was subsequently performed on all isolates confirmed to be *E*. *coli*.

### Phenotypic antimicrobial susceptibility testing

Antimicrobial susceptibility testing was performed on *E*. *coli* isolates according to the European Committee on Antimicrobial Susceptibility Testing (EUCAST) disc diffusion method using the following antibiotic discs, selected to represent commonly used agents for *E*. *coli* infections in human and veterinary medicine: Nalidixic acid (30 µg), Nitrofurantoin (100 µg), Piperazillin-tazobactam (36 µg), Tetracycline (30 µg), Trimethoprim (5 µg), Trimethoprim-sulfamethoxazole (25 µg), Meropenem (10 µg), Ciprofloxacin (5 µg), Ampicillin (10 µg), Cefadroxil (30 µg), Chloramphenicol (30 µg), Gentamicin (10 µg) and Mecillinam (10 µg) (Thermo Fisher Scientific Oxoid Ltd, Hants, UK). The inhibition zone diameters were interpreted according to EUCAST breakpoints^65^, or, for antibiotics with no defined clinical breakpoints (i.e. Nalidixic acid, Tetracycline and Chloramphenicol), the inhibition zone diameters were interpreted by breakpoints defined by the Normalized Resistance Interpretation method^66^.

Phenotypic characterization to identify ESBL-producing isolates was performed using the following five antibiotic discs: Ceftazidime (10 µg), Cefotaxime (5 µg), Cefepime (30 µg), Cefoxitin (30 µg) and Amoxicillin/clavulanic acid (30/1 µg) (Thermo Fisher Scientific Oxoid Ltd, Hants, UK). Inhibition zone diameters were used to determine the phenotypes, according to EUCAST guidelines^67^.

### Library preparation and whole genome sequencing

DNA was extracted from all ESBL-producing *E*. *coli* isolates using the MagnaPure compact nucleic acid isolation kit (Roche, Mannheim, Germany). Multiplexed DNA libraries were prepared using the NexteraXT library preparation kit (Illumina, San Diego, USA) according to manufacturer’s instructions. Paired-end WGS was performed using the MiSeq platform (Illumina, San Diego, USA) using either 250 or 300 base pair read lengths. Average read depth was determined by reference mapping to the *E*. *coli* K12 genome (NC_000913.3).

### *De novo* assembly

ConDeTri was used to remove reads with low quality scores, trim high quality reads and remove duplicate reads^68^. High-quality, trimmed, unique reads were assembled with SPAdes^69^ using default parameters and k-values of 21, 33, 55, 77, 99, and 127. A second assembly excluding the k-value 127 was also performed to improve assemblies with shorter read lengths. Assembly quality was evaluated with QUAST^70^ and the assembly with the fewest number of contigs and highest N50 was used in downstream analyses. Additionally, PlasmidSPAdes was implemented with default settings to detect and assemble putative plasmid sequences from trimmed reads^71^. See File S1 for specific commands and settings used for each program.

### Core genome analysis

An alignment of orthologous sequences conserved in all genomes was generated from *de novo* assemblies using Parsnp^72^, with the –c option invoked to force inclusion of all genomes. Reference genomes *E*. *fergusonii* (NC_011740.1) and *E*. *coli* TW10509 (NZ_GL872204.1) were included to provide phylogenetic context for divergent strains. The resulting core genome alignment and SNP tree was used as input into ClonalFrameML^73^, with default settings, to detect recombination and reconstruct the phylogeny based on an evolutionary model that accounts for both mutation and recombination events. The previously described methodology was re-performed excluding reference and divergent genomes to improve phylogenetic resolution of the 25 most closely related isolates. *In silico* phylotyping, based on the Clermont *et al*. typing scheme^74^, was performed using a previously described methodology^75^. The program SRST2 (v0.2.0)^76^ was used to identify MLST types by matching reads to the *Escherichia coli* #1 database^77^ downloaded from pubmlst.org.

### Accessory genome analysis

AMR genes were detected from trimmed, unique high quality reads using SRST2^76^ and matched to the ARGannot resistance gene database^78^. This manually curated AMR gene database includes sequences found in both the ResFinder database^79^ and the Comprehensive Antibiotic Resistance Database (CARD)^80^, and includes both acquired resistance genes and point mutations in chromosomal target genes. AMR gene sequences from each isolate were extracted using the “report_new_consensus” function in SRST2, which outputs all reads mapped to the gene sequence. Gene alignments were visualized in Geneious (v10.1.3)^81^ and insertions/deletions (indels) were confirmed or rejected by *de novo* assembly of mapped reads. Sequences differing by one or more confirmed SNP or indel were considered a unique allele. Maximum likelihood phylogenetic trees of AMR genes with more than two alleles were estimated in the statistical programming language R (v3.3.3)^82^ using the package phangorn (v2.2.0)^83^. Separate nucleotide substitution model tests^84^ were performed on each gene alignment and the model with the lowest Akaike information criterion value was used to estimate phylogenies with 100 bootstrap pseudoreplicates. AMR gene presence was mapped onto the core genome phylogeny using the -phydataplot function in the R package ape (v4.1)^85^, with different colours representing different alleles. SRST2-detected AMR genes were queried and visualized against putative plasmid contigs that were assembled with PlasmidSPAdes from select isolates using Bandage^86^. Biocide and metal resistance genes were detected from assembled contigs using BacMetScan^87^, using the experimentally confirmed resistance genes dataset (v1.1), and visualized on putative plasmid contigs using Bandage, as described above. Predicted resistance to particular compounds and gene location (chromosomal or plasmid) was obtained from the BacMet database. *In silico* PCR amplification of the class 1 integron-integrase gene *intI1* was performed using the program seqpoet (v0.3.4) using previously described primers (IntiIf: TTCGAATGTCGTAACCGC and IntiIr: CGAGGCATAGACTGTAC)^88^ and the SPAdes-assembled contigs.

Accessory genomes were further explored using the program LS-BSR^89^ to determine the relative level of relatedness among isolates of each detected coding sequence (CDS). Core sequences were excluded using the filter_BSR_variome script. Accessory CDS with BLAST score ratio (BSR) values ≥0.70 were considered present and a binary present/absent matrix was created as input for the Bayesian clustering tool K-Pax2^90^ to partition isolates based on their accessory genome content. Default prior settings were used with an initial partition of 26 units (one for each isolate). All data that support the findings of this publication can be found in Ahlstrom *et al*.^91^.

### Data Availability

Raw WGS reads have been deposited in the Sequence Read Archive; accession number: SRP126755.

## Electronic supplementary material

Supplementary material
