# Supplementary material for: Acquisition and dissemination of cephalosporin-resistant E. coli in migratory birds sampled at an Alaska landfill as inferred through genomic analysis
Source: Sci Rep. 2018 May 9;8:7361. doi: 10.1038/s41598-018-25474-w (PMC5943298; doi:10.1038/s41598-018-25474-w)
Supplement: Supplementary file 1 — Supplementary material [file 41598_2018_25474_MOESM1_ESM.pdf]

Supplementary material

Acquisition and dissemination of cephalosporin-resistant *E. coli* in migratory birds sampled at an Alaska landfill as inferred through genomic analysis

Christina A. Ahlstrom[a]*, Jonas Bonnedahl[b,c], Hanna Woksepp[d], Jorge Hernandez[e], Björn Olsen[f,g], Andrew M. Ramey[a]*

[a]U.S. Geological Survey, Alaska Science Center, Anchorage, Alaska, 99508, USA
[b]Centre for Ecology and Evolution in Microbial Model Systems, Linnaeus University, SE - 39182, Sweden
[c]Department of Infectious Diseases, Kalmar County Hospital, Kalmar, SE-39185, Sweden
[d]Department of Clinical Microbiology, Kalmar County Hospital, Kalmar, SE-39185, Sweden
[e]Department of Microbiology, Kalmar County Hospital, Kalmar, SE-39185, Sweden
[f]Zoonosis Science Center, Department of Medical Biochemistry and Microbiology, Uppsala University, Uppsala, SE-75185, Sweden
[g]Section for Infectious Diseases, Department of Medical Sciences, Uppsala University, Uppsala, SE-75185, Sweden

*Address correspondence to Christina A. Ahlstrom, cahlstrom@usgs.gov or Andrew M. Ramey, aramey@usgs.gov.

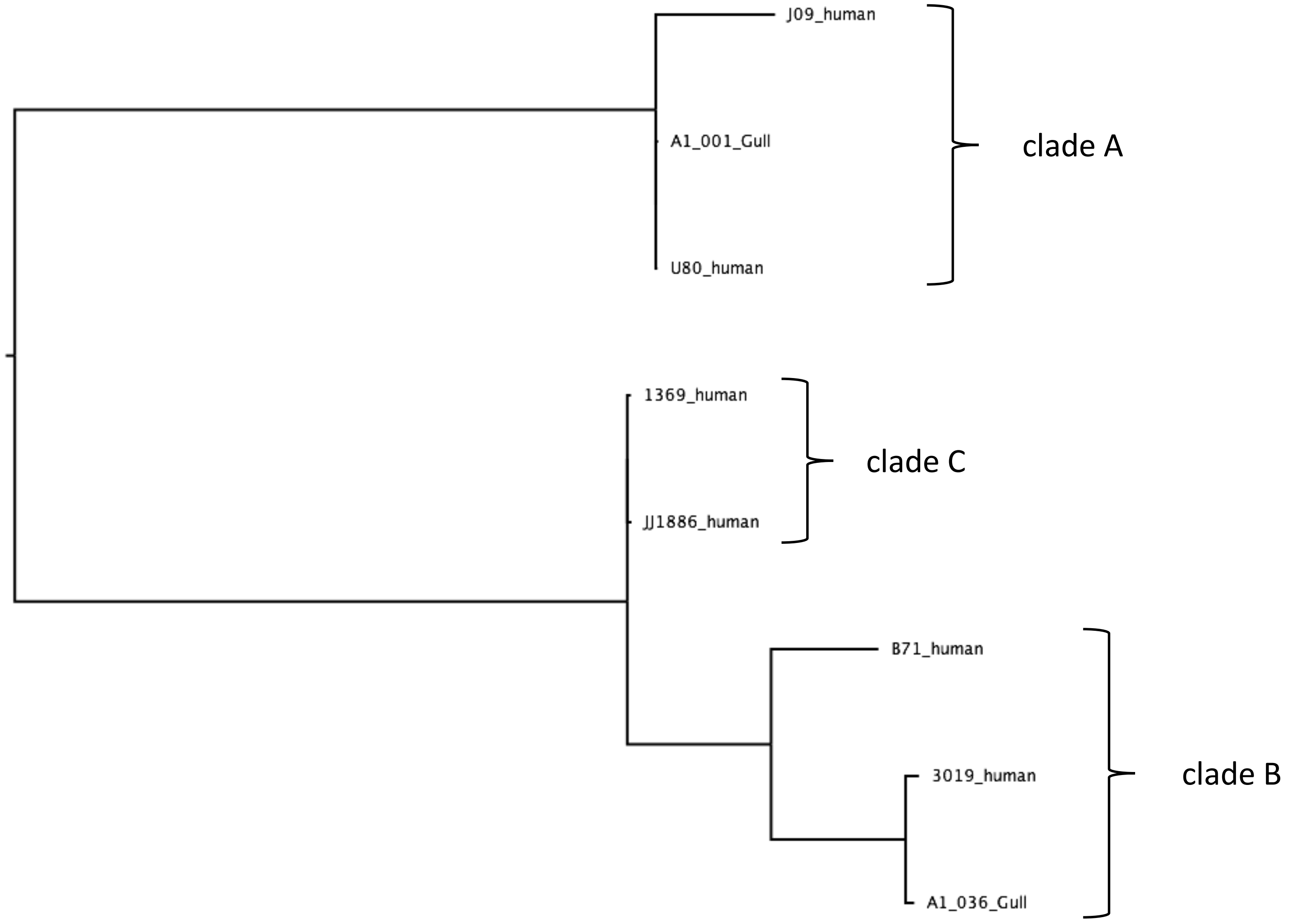

Fig. S1 Midpoint rooted clonal core genome phylogeny of ST131 *E. coli* isolates isolated from large-bodied gulls in this study and human clinical samples previously reported representing the three ST131 clades (McNalley et al., 2016).

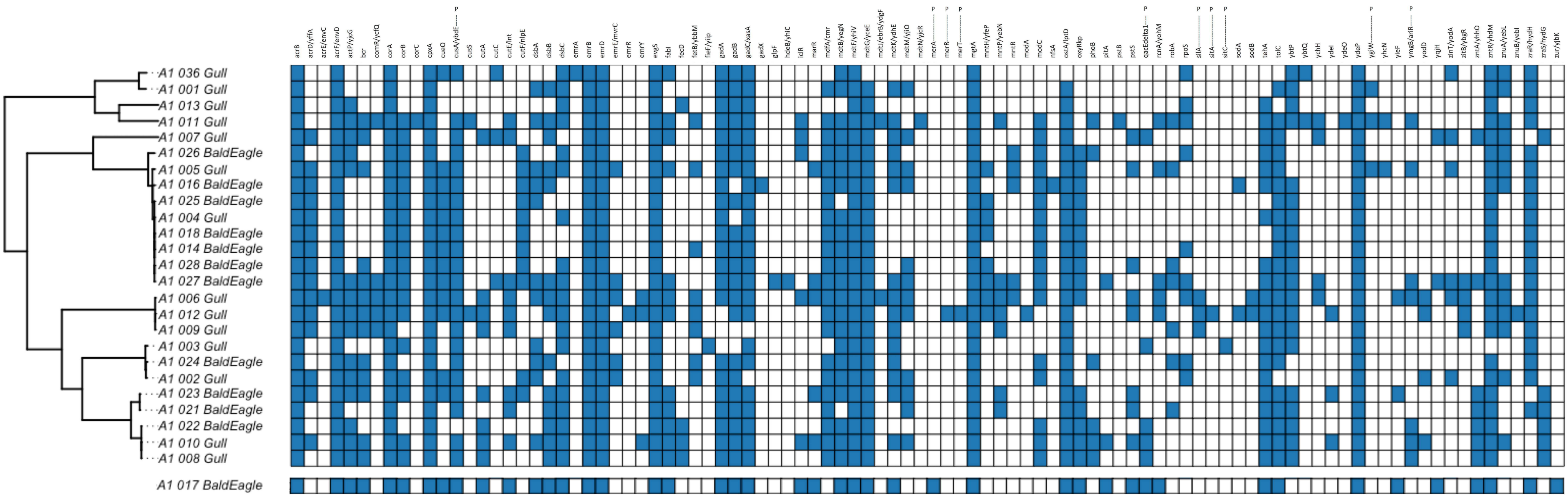

Fig. S2 Midpoint rooted clonal core genome phylogeny of 25 ESBL-producing *E. coli* isolates originating from large-bodied gulls and bald eagles in Alaska. Presence of each of 96 BacMet-identified biocide/heavy metal resistance genes is shown as a matrix, with colored squares indicating presence and white indicating that the gene was not found. The divergent A1_017_BaldEagle isolate is included at the bottom of the matrix. The letter "P" above the gene names indicates the gene has previously been found to be plasmid-borne.

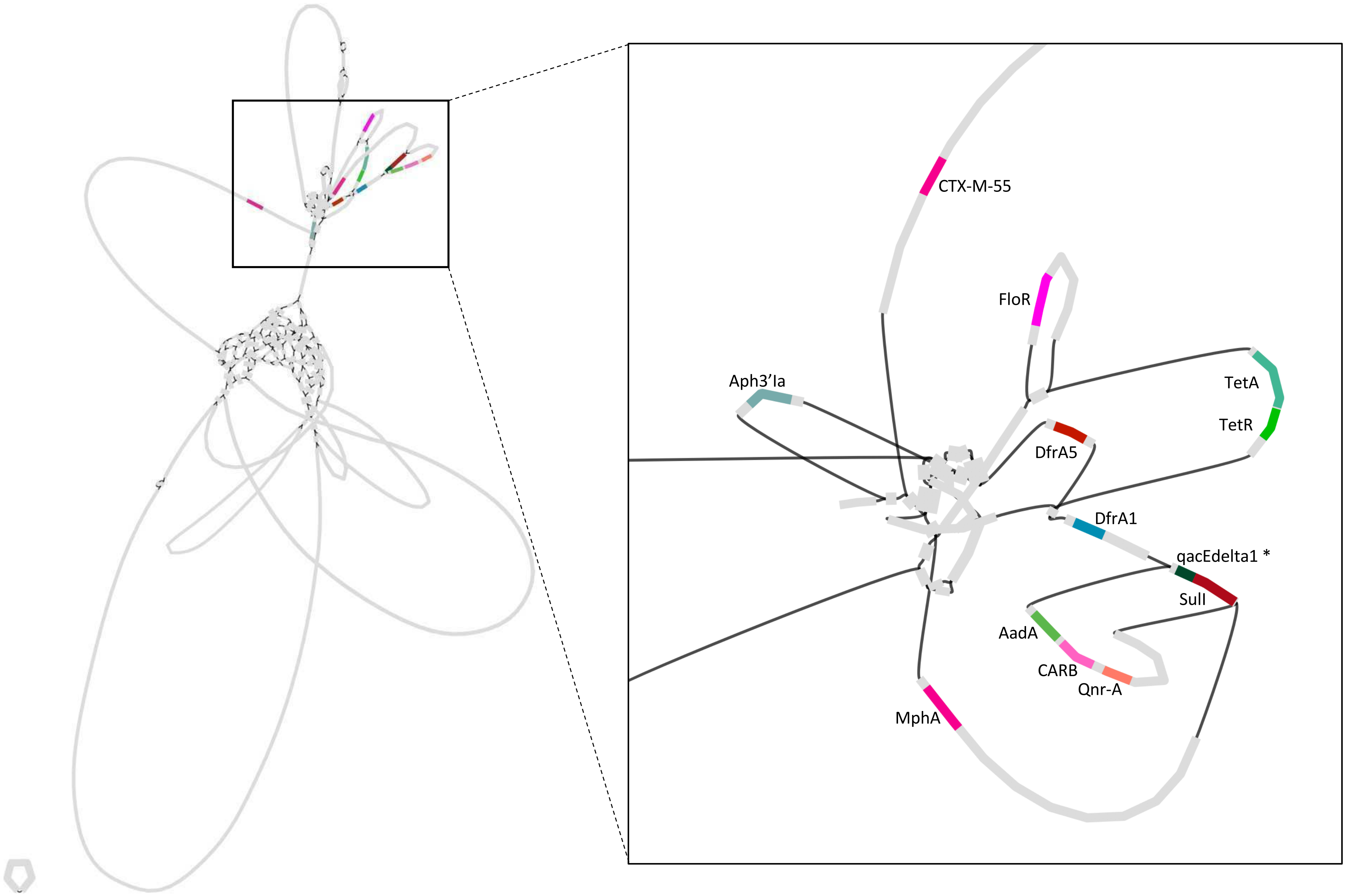

Fig. S3 Isolate A1_007_Gull putative plasmid contigs (grey rectangles) assembled by PlasmidSPAdes and visualized with Bandage. Likely connections between contigs are shown by black lines. The locations of AMR genes and biocide/heavy metal resistance genes are indicated by colored rectangles, labeled with the gene name. Biocide/heavy metal resistance genes are indicated by an asterisk (*).

Supplementary Table 1 Phenotypic resistance profiles to 18 antibiotic discs of ESBL-producing *E. coli* isolates originating from large-bodied gull and bald eagle fecal samples in Alaska.

Isolate ID	NAL	NIT100	PTZ36	TET	TRI5	TSU25	MER10	CIP5	AMP10	CDR30	KLO	GEN10	MEC10	AMC	FEP	FOX	CFZ	CTX
**A1_001_Gull**	S	S	S	S	S	S	S	S	R	R	S	S	S	R	R	R	S	R
**A1_002_Gull**	S	S	S	S	S	S	S	S	R	R	S	S	S	S	R	S	S	R
**A1_003_Gull**	S	S	S	R	S	S	S	S	R	R	S	S	S	R	S	R	R	S
**A1_004_Gull**	S	S	R	S	S	S	S	S	R	R	S	S	S	R	S	R	R	S
**A1_005_Gull**	S	S	I	S	S	S	S	S	R	R	S	S	S	R	S	R	R	S
**A1_006_Gull**	S	S	S	R	S	S	S	S	R	R	S	S	S	R	S	R	S	S
**A1_007_Gull**	R	R	S	R	R	S	S	R	R	R	R	S	S	R	R	R	R	R
**A1_008_Gull**	S	S	S	S	R	R	S	S	R	R	S	S	S	R	S	R	R	S
**A1_009_Gull**	S	S	I	R	S	S	S	S	R	R	S	S	S	R	S	R	S	S
**A1_010_Gull**	S	S	S	S	R	R	S	S	R	R	S	S	S	R	S	R	R	S
**A1_011_Gull**	S	S	I	S	S	S	S	S	R	R	S	S	S	R	S	R	R	R
**A1_012_Gull**	S	S	S	R	S	S	S	S	R	R	S	S	S	R	S	R	S	S
**A1_013_Gull**	S	S	S	S	S	S	S	S	R	R	S	S	S	R	S	R	S	S
**A1_014_BaldEagle**	S	S	R	S	S	S	S	S	R	R	S	S	S	R	S	R	I	S
**A1_016_BaldEagle**	S	S	I	R	S	S	S	S	R	R	S	S	S	R	S	R	R	R
**A1_017_BaldEagle**	S	S	S	R	S	S	S	S	R	R	S	S	S	R	S	R	R	R
**A1_018_BaldEagle**	S	S	R	S	S	S	S	S	R	R	S	S	S	R	S	R	I	S
**A1_021_BaldEagle**	S	S	S	R	S	S	S	S	R	R	S	S	S	R	S	R	R	R
**A1_022_BaldEagle**	S	S	R	S	S	I	S	S	R	R	S	S	S	R	S	R	R	R
**A1_023_BaldEagle**	S	S	I	S	R	R	S	S	R	R	S	S	S	R	S	R	R	S
**A1_024_BaldEagle**	S	S	S	S	S	S	S	S	R	R	S	S	S	R	S	R	R	R
**A1_025_BaldEagle**	S	S	S	S	S	S	S	S	R	R	S	S	S	R	S	R	R	S
**A1_026_BaldEagle**	S	S	R	S	S	S	S	S	R	R	S	S	S	R	S	R	I	S
**A1_027_BaldEagle**	S	S	I	S	S	S	S	S	R	R	S	S	S	R	S	R	S	S
**A1_028_BaldEagle**	S	S	R	S	S	S	S	S	R	R	S	S	S	R	S	R	I	S
**A1_036_Gull**	S	S	R	S	S	S	S	S	R	R	S	S	S	R	S	R	R	S

S = Susceptible, I = Intermediate, R = Resistant

NAL = nalidixic acid, NIT100 = nitrofurantoin, PTZ36 = piperacillin-tazobactam, TET = tetracycline, TRI5 = trimethoprim, TSU25 = trimethoprim-sulfamethoxazole, MER10 = meropenem, CIP5 = ciprofloxacin, AMP10 = ampicillin, CDR30 = cefadroxil, KLO = chloramphenicol, GEN10 = gentamicin, MEC10 = mecillinam, AMC = Amoxicillin/clavulanic acid, FEP = Cefepime, FOX = Cefoxitin, CFZ = Ceftazidime, CTX = Cefotaxime

Supplementary Table 2 WGS *de novo* assembly metrics of 27 ESBL-producing *E. coli* isolates originating from large bodied gull and bald eagle fecal samples in Alaska.

Isolate ID	mean # contigs >500bp
**A1_001_Gull**	104
**A1_002_Gull**	81
**A1_003_Gull**	84
**A1_004_Gull**	82
**A1_005_Gull**	179
**A1_006_Gull**	206
**A1_007_Gull**	108
**A1_008_Gull**	146
**A1_009_Gull**	90
**A1_010_Gull**	241
**A1_011_Gull**	170
**A1_012_Gull**	372
**A1_013_Gull**	89
**A1_014_BaldEagle**	74
**A1_016_BaldEagle**	120
**A1_017_BaldEagle**	107
**A1_018_BaldEagle**	100
**A1_020_BaldEagle**	931
**A1_021_BaldEagle**	139
**A1_022_BaldEagle**	263
**A1_023_BaldEagle**	231
**A1_024_BaldEagle**	212
**A1_025_BaldEagle**	92
**A1_026_BaldEagle**	160
**A1_027_BaldEagle**	256
**A1_028_BaldEagle**	122
**A1_036_Gull**	52

File S1: Command line arguments for programs used

Arguments:
$1= prefix for each isolate ID
$2= path to SPAdes-assembled contigs

# ConDeTri
perl condetri.pl -fastq1=$1_R1.fastq -fastq2=$1_R2.fastq -prefix=$1 -sc=33

perl filterPCRdupl.pl -fastq1=$1_trim1.fastq -fastq2=$1_trim2.fastq -prefix=$1

# SPAdes
python spades.py -k 21,33,55,77,99,127 --cov-cutoff 'auto' --careful -1 $1_uniq1.fastq -2 $1_uniq2.fastq

# PlasmidSPAdes
python spades.py -k 21,33,55,77,99,127 --cov-cutoff 'auto' --plasmid --careful -1 $1_uniq1.fastq -2 $1_uniq2.fastq

# Parsnp
parsnp -r ! -d $2 -c

# ClonalFrameML
./ClonalFrameML "path to parsnp nwk tree file" "path to parsnp xmfa file" -xmfa_file true

# in silico phylotyping (Kaas et al., 2012)
perl Ks_phylotyper.pl -t Phylotyping_products.txt -r rules_table.txt -l results.txt $2

# SRST2 MLST typing
python srst2.py --input_pe $1_uniq1.fastq $1_uniq2.fastq --log --mlst_db Escherichia_coli1.fasta --mlst_definitions ecoli.txt --forward _uniq1 --reverse _uniq2

# SRST2 AMR gene detection
python srst2.py --input_pe $1_uniq1.fastq $1_uniq2.fastq --log --gene_db srst2/data/ARGannot.r1.fasta --forward _uniq1 --reverse _uniq2 --report_all_consensus

python srst2.py --prev_output "path to srst2 AMR gene detection results" --output compiled_AMR_results

python consensus_alignment.py --in "path to srst2 AMR gene detection results" *.all_consensus_alleles.fasta --type gene

# BacMet biocide/heavy metal resistance gene detection
perl BacMet-Scan.pl -i $2 -d BacMet_EXP.704

# Seqpoet *in silico* PCR
seqpoet --pcr --out $1_insilicoPCR_results.fasta $2.fasta primers.txt

# LS-BSR
python ls_bsr.py -d $2 -c usearch

python filter_BSR_variome.py --bsr_matrix= bsr_matrix.txt
